# Identification of *SCN1A* and *PCDH19* Mutations in Chinese Children with Dravet Syndrome

**DOI:** 10.1371/journal.pone.0041802

**Published:** 2012-07-25

**Authors:** Anna Ka-Yee Kwong, Cheuk-Wing Fung, Siu-Yuen Chan, Virginia Chun-Nei Wong

**Affiliations:** Department of Paediatrics and Adolescent Medicine, Li Ka Shing Faculty of Medicine, The University of Hong Kong, Hong Kong SAR, China; Charité Universitätsmedizin Berlin, NeuroCure Clinical Research Center, Germany

## Abstract

**Background:**

Dravet syndrome is a severe form of epilepsy. Majority of patients have a mutation in *SCN1A* gene, which encodes a voltage-gated sodium channel. A recent study has demonstrated that 16% of *SCN1A*-negative patients have a mutation in *PCDH19*, the gene encoding protocadherin-19. Mutations in other genes account for only a very small proportion of families. *TSPYL4* is a novel candidate gene within the locus 6q16.3-q22.31 identified by linkage study.

**Objective:**

The present study examined the mutations in epileptic Chinese children with emphasis on Dravet syndrome.

**Methods:**

A hundred children with severe epilepsy were divided into Dravet syndrome and non-Dravet syndrome groups and screened for *SCN1A* mutations by direct sequencing. *SCN1A*-negative Dravet syndrome patients and patients with phenotypes resembling Dravet syndrome were checked for *PCDH19* and *TSPYL4* mutations.

**Results:**

Eighteen patients (9 males, 9 females) were diagnosed to have Dravet syndrome. Among them, 83% (15/18) had *SCN1A* mutations including truncating (7), splice site (2) and missense mutations (6). The truncating/splice site mutations were associated with moderate to severe degree of intellectual disability (p<0.05). During the progression of disease, 73% (11/15) had features fitting into the diagnostic criteria of autism spectrum disorder and 53% (8/15) had history of vaccination-induced seizures. A novel *PCDH19* p.D377N mutation was identified in one *SCN1A*-negative female patient with Dravet syndrome and a known *PCDH19* p.N340S mutation in a female non-Dravet syndrome patient. The former also inherited a *TSPYL4* p.G60R variant.

**Conclusion:**

A high percentage of *SCN1A* mutations was identified in our Chinese cohort of Dravet syndrome patients but none in the rest of patients. We demonstrated that truncating/splice site mutations were linked to moderate to severe intellectual disability in these patients. A *de novo PCDH19* missense mutation together with an inherited *TSPYL4* missense variant were identified in a patient with Dravet syndrome.

## Introduction

*SCN1A* is regarded as the most important epilepsy gene associated with a spectrum of epilepsy syndromes ranging from milder phenotypes in generalized epilepsy with febrile seizures plus (GEFS+) to severe myoclonic epilepsy of infancy (Dravet syndrome).

GEFS+ is inherited in an autosomal-dominant pattern [1]. It includes a spectrum of phenotypes with mild and severe forms of epilepsy associated with febrile and afebrile seizure. The most severe form in GEFS+ spectrum is Dravet syndrome (DS) [2]. DS is defined by febrile and afebrile generalized and unilateral, clonic or tonic-clonic seizures which begin in the first year of life [3]. The seizure begins in form of prolonged febrile seizures which may evolve to status epilepticus [1], [4], [5]. Other seizure types such as myoclonic, focal, absence and atonic seizures emerge later. Development is normal in the first year but delayed from the second year of life followed by cognitive and personality impairment. Pyramidal signs and ataxia may evolve. The overall syndrome in DS is resistant to antiepileptic drugs.

*SCN1A* is the gene encoding the α1 pore-forming subunit of voltage-gated sodium channel which plays an important role for initiating and propagating action potentials in the central nervous system [6]. This subunit consists of four homologous domains (D1–D4) and each domain contains 6 transmembrane segments (S1–S6) [7]. To date, more than 600 *SCN1A* variants have been identified throughout the whole gene [8]. These mutations may alter the function of the channel leading to abnormal hyper-excitability of neural network and resulting in epileptic seizure [9]. Although *SCN1A* mutations were initially identified in GEFS+ families [7], most of the mutations were found in DS patients. Majority (70–80%) of DS patients were found to have a point mutation or gross rearrangement in their *SCN1A* gene [10], [11], [12]. The remaining 20–30% of the patients presented with the same severe phenotype of a high rate of epilepsy. Mutations in other genes have been reported in these patients [13].

Mutations in *PCDH19* have been reported in patients with epilepsy and mental retardation limited to females (EFMR), which is X-linked and connected through unaffected transmitting males [14], [15], [16]. *PCDH19* encodes protocadherin-19, a protein belonging to the delta-2 protocadherin subclass of the cadherin superfamily. It is a calcium-dependent cell-cell adhesion protein primarily expressed in the nervous system and is involved in neuronal connections and signal transductions at the synaptic membrane [17], [18], [19], [20]. EFMR can be differentiated from DS with clinical features being highly variable with the onset of seizures ranging from 6 to 36 months, combination of febrile and afebrile seizures and variable degree of psychomotor delay and cognitive impairment [21], [22]. In a recent study, Depienne et al [22] identified point mutations in *PCDH19* genes in a group of *SCN1A*-negative patients with DS. They found that 16% of *SCN1A*-negative DS patients were *PCDH19*-positive and *PCDH19* might overall account for 5% of DS patients. Although the clinical features of *PCDH19*-DS patients were slightly different from the classical *SCN1A*-DS patients, their results demonstrated that *PCDH19* was involved in epileptic encephalopathies clinically overlapping with DS.

Mutations in two other genes encoding sodium channel subunits (*SCN1B*, *SCN2A*) and 2 genes encoding GABA receptor subunits (*GABRG2* and *GABRD*) have been found in a few GEFS+ families. The discovery of more susceptibility genes is important for the improvement of genetic diagnosis and management of epilepsy syndromes. In a recent study, computational disease gene prioritization based on expression pattern in Human Brain Gene Expression Atlases was applied to evaluate the candidate genes in previously mapped novel susceptible GEFS+ locus 6q16.3-q22.31 [23], [24]. *TSPYL1* and *TSPYL4* genes, two members of nucleosome assembly protein, were found to be outstanding candidates for GEFS+. As nucleosome assembly protein can regulate gene expression and may present a novel mechanism for disease, we have investigated *TSPYL1* and *TSPYL4* sequence variations in this study.

In the present study, *SCN1A* mutation analysis was performed in 100 patients recruited from the Epilepsy Clinic of Queen Mary Hospital and Duchess of Kent Children’s Hospital, being affiliated hospitals of the University of Hong Kong. The cohort was divided into two groups. The first group included 18 patients who developed severe epilepsies and were diagnosed clinically to have DS. The second group consisted of 82 patients with other epileptic phenotypes who did not fit into the diagnostic criteria of DS. We aim to study the rate of *SCN1A* mutations in these two groups of patients and further investigate the association between *SCN1A* mutation and phenotype. In addition, mutations in *TSPYL4* and *PCDH19* were identified in two patients, with one of them carrying both *PCDH19* and *TSPYL4* mutations.

## Methods

### Ethics Statement

This study was approved by the Institutional Review Board of the Hong Kong West Cluster and the University of Hong Kong (IRB Ref. No.: UW 11-190). Written informed consent was obtained from the parents of children involved in the study since 2011. Prior to 2011, documented verbal consent was obtained from the subjects’ parents. All data were analyzed anonymously.

### Patient Samples and Clinical Diagnoses

One hundred patients with epilepsy being actively followed up in the Epilepsy Clinic of Queen Mary Hospital and Duchess of Kent Children’s Hospital were recruited. Informed consent was obtained from their parents. Clinical data including the age of onset, seizure types, precipitating factors, developmental course, and family history of seizures, imaging and EEG reports were evaluated retrospectively. Children with autistic features were diagnosed to be autism spectrum disorder (ASD) based on DSM IV TR diagnostic criteria. Patients with intellectual disability (ID) were assessed according to their scores of general/developmental quotients as mild ID (general quotient = 50–70), moderate ID (general quotient = 25–50) or severe ID (general quotient<25). The DS patients were identified based on International League Against Epilepsy (ILAE) classification (1981, 1989) and core DS phenotypes defined by Dravet [3] including normal development before onset of seizure, onset in the first year of age by febrile or afebrile clonic and tonic-clonic, generalized and unilateral seizure, often prolonged, subsequent appearance of multiple seizure types including myoclonic, atypical absences and focal seizures and delay in developmental and cognitive skills. DS included both severe myoclonic epilepsy of infancy and borderline severe myoclonic epilepsy of infancy [5]. Both the 18 DS patients and the remaining 82 non-DS patients were screened for *SCN1A* mutations. Among the non-DS patients, 25 patients (12 male, 13 female) with clinical features resembling DS but varying in two criteria from DS phenotypes such as late seizure onset, less intractable seizure, absence of status epilepticus, myoclonic jerks or atypical absences were selected for *PCDH19* and *TSPYL4* mutation study.

Control DNA samples were extracted from blood collected from Hong Kong Red Cross. A hundred control alleles were sequenced to confirm the mutations detected in the present study were not polymorphisms.

### Molecular Analysis of *SCN1A* Gene

#### Polymerase chain reaction

Genomic DNA samples of the 100 patients were extracted from peripheral blood using Flexigene DNA Kit (Qiagen GmbH, Germany). All 26 exons of *SCN1A* including the splice junctions were amplified by polymerase chain reaction (PCR) using oligonucleotide primers designed based on the sequence of *SCN1A* gene on human chromosome 2 (GeneBank accession number: NC_000002.11). PCR contained 0.1 µg of genomic DNA as template, 5 pmol of each primer, 200 µM of deoxyribonucleoside triphosphates, and 0.5 U HotStarTaq Plus DNA Polymerase (Qiagen) in 1X Qiagen PCR buffer. PCR was carried out with initial enzyme activation at 95°C for 5 minutes, followed by 50 cycles of denaturation at 94°C for 30 seconds, annealing at 60°C for 1 minute and extension at 72°C for 1.5 minutes, with a final extension at 72°C for 10 minutes. The quality and quantity of PCR products were checked by electrophoresis.

#### DNA sequencing

PCR products were sequenced by Bigdye Terminator v3.1 Cycle Sequencing Kit (Applied Biosystem, Foster City, CA) using either the original PCR primers or nested intron flanking primers. Sequences were analyzed on a 3730xl sequencer (Applied Biosystems). Homology analyses with *SCN1A* reference sequence were performed using NCBI program BLAST®. The numbering for each mutation was taken from the start codon with +1 corresponding to the A of the ATG in the reference sequence of *SCN1A* isoform 1 (Genbank accession number: NM_001165963). *SCN1A* mutations or sequence variants were discriminated from single nucleotide polymorphisms (SNP) reported in NCBI SNP and Ensembl SNP database. Novel mutations were verified by checking the already identified *SCN1A* variants in the *SCN1A* Variant Database [8]. The parental DNA was collected and sequenced to distinguish between *de novo* and familial variants.

#### Pathogenicity assessment of mutation

Several analyses were used to evaluate the pathogenicity of the missense mutation. Firstly, conservative analyses were performed to predict whether the amino acid substitutions in missense mutations affect protein function based on the degree of conservation at the affected residues. Human *SCN1A* amino acid sequences were aligned with the paralogous and orthologous sequences by an online program ClustalW2 Multiple Sequence Alignment (European Bioinformatics Institute, EMBI, UK). The GenBank accession numbers for amino acid alignments were NP_001159435 for human SCN1A, NP_001035232 for human SCN2A, NP_008853 for human SCN3A, NP_000325 for human SCN4A, NP_055006 for human SCN8A, NP_002968 for human SCN9A, XP_001154158 for chimpanzee SCN1A, XP_001100928 for rhesus monkey SCN2A, NP_061203 for mouse SCN1A, NP_110502 for rat SCN1A, XP_001252710 for cattle SCN1A, XP_422021 for fowl SCN1A, and CAQ13572 for zebrafish SCN1A. Secondly, the mutations were analyzed by an online sequence homology-based tool, Sorting Intolerant from Tolerant (SIFT) (Genome Institute of Singapore, http://sift.bii.a-star.edu.sg/) [25]. Thirdly, a web-based program Align-GVGD (http://agvgd.iarc.fr/agvgd_input.php) [26], was used to predict whether the mutations are deleterious to protein functions by combining the biophysical properties of amino acids and multiple sequence alignment [27]. We applied the multiple sequence alignment used in our conservative analysis to Align-GVGD. Grantham Variation determined the range of toleration at a specific amino acid position and Grantham Deviation was the physiochemical difference between the wild type and mutated amino acid. The variants were classified into 7 groups (class 0, class 15, class 25, class 35, class 45, class 55 and class 65) in the program ranging from least likely (class 0) to most likely (class 65) to interfere the protein function. The two mutations in intron regions close to splice site were analyzed by another online software tools, Automated Splice Site Analyses (Laboratory of Human Molecular Genetics and Genomic Disorders, UWO, CA, https://splice.uwo.ca/) [28]. The functional domains in the amino acid sequence were defined by the database UniProt with accession number P35498.

### Molecular Analysis of *PCDH19* Gene

Mutations of *PCDH19* genes were screened in the 3 *SCN1A*-negative DS patients and 13 female non-DS patients with clinical features resembling DS. The 6 exons of *PCDH19* gene covering the coding regions as well as the splice junctions were amplified by PCR using oligonucleotide primers designed based on the sequence of *PCDH19* gene on human chromosome X (GeneBank accession number: NG_021319). PCR, DNA sequencing, homology analysis and pathogenicity assessment were performed as mentioned above. GenBank accession numbers for amino acid alignments were Q8TAB3 for human PCDH19, AAI11561 for human PCDH10, CAI17092 for human PCDH17, AAI43362 for human PCDH18, XP_003317603 for chimpanzee PCDH19, XP_001091349 for rhesus monkey PCDH19, Q80TF3 for mouse PCDH19, NP001162600 for rat PCDH19, DAA13144 for cattle PCDH19, and NP_001120991 for zebrafish PCDH19. The mutation nomenclature was based on the *PCDH19* transcript reference sequence (GeneBank accession number: NM_020766) and nucleotides were numbered according to the cDNA with +1 corresponding to the A of the ATG translation initiation codon in the reference sequence. The functional domains in the amino acid sequence were defined by the database UniProt with accession number Q8TAB3.

### Molecular Analysis of *TSPYL1* and *TSPYL4* Genes

Polymorphisms in both genes were extracted from the dbSNP database. Mutations of *TSPYL4* gene were checked in the 3 *SCN1A*-negative DS patients and 25 non-DS patients with clinical features resembling DS. The coding sequence of *TSPYL4* was amplified by PCR using primers designed based on the reference sequence of *TSPYL4* gene on human chromosome 6 (GeneBank accession number: NC_000006). PCR, DNA sequencing, homology analysis and pathogenicity assessment were performed as mentioned above. GenBank accession numbers for amino acid alignments were NP_067680 for human TSPYL4, NP_003300 for human TSPYL1, XP_518704 for chimpanzee TSPYL4, NP_001181803 for rhesus monkey TSPYL4, NP_001094534 for cattle TSPYL4, NP_084479 for mouse TSPYL4, and NP_001012075 for rat TSPYL4. The mutation nomenclature was based on the reference sequence (GeneBank accession no.: NM_021648) and nucleotides were numbered according to the cDNA with +1 corresponding to the A of the ATG translation initiation codon.

### Statistical Analysis

Chi-square test was employed to illustrate the relationship between types of *SCN1A* mutations (missense mutation versus truncating/splice site mutation) and the degree of ID of the patients (normal to mild ID versus moderate to severe ID). Statistical analysis was performed by statistical program PRISM V.5.0 (GraphPad Software Inc., San Diego, CA).

## Results

### Clinical Diagnoses (Table 1)

Eighteen patients (9 males, 9 females) were diagnosed to have DS. All DS patients had normal development before seizure onset and had onset before one year of age with an average onset age of 5.4 months. All patients experienced febrile seizures and had three or more types of seizures such as partial, generalized tonic-clonic, tonic, clonic, absence and myclonic seizures. Prolonged seizures (>10 min) or status epilepticus were common and 16 DS patients had different grades of intellectual disabilities. Twelve patients (12/18; 66%) had autistic features compatible to the clinical diagnosis of Autism Spectrum Disorder. Eight patients (8/18; 44%) had a history of vaccination induced seizures. The remaining 82 patients were classified as non-DS patients as their clinical features could not fulfill the diagnostic criteria of DS.

**Table 1:** Summary of clinical characteristics of 18 patients with Dravet syndrome and 1 *PCDH19*-positive patient not grouped under Dravet syndrome.

Case	Sex	Age (yr.)	Age of seizureonset (mo.)	Febrile seizure	Seizure types				Prolonged seizures/status epilepticus	Intellectual disability	ASD	Vaccine induced	*SCN1A* mutation (other mutations)
					Partial	Gener-alized tonic-clonic	Myo-clonic	Absences					
6	F	15	6	+	+	+	+	−	+	Moderate	+	−	Truncation
24	F	18	10	+	+	+	+	+	+	Moderate	+	−	Splice site
40	F	11	5	+	+	+	+	+	−	Mild	+	−	Missense
45	M	9	7	+	+	+	+	−	−	Normal (limited intelligence)	+	−	−
46	M	10	4	+	+	+	+	+	+	Mild	+	DPT (onset)	Missense
53	F	8	5	+	+	+	+	+	+	Normal (learning problem)	+	Flu vaccine	Truncation
60	F	5	Day 3–4	+	+	+	+	−	−	Severe	−	−	−
65	M	12	8	+	+	+	+	+	+	Moderate	−	DPT	Missense
71	F	17	8	+	+	+	+	−	−	Severe	+	−	Truncation
74	M	18	8	+	+	+	+	+	+	Severe	+	−	Truncation
75	F	3	6	+	+	+	+	−	+	Mild	−	DPT	Missense
76	M	3	3	+	+	+	+	+	+	Mild	+	5 in 1 (onset)	Missense
77	M	Died at 3 yr	4	+	+	+	−	+	+	Mild	−	DPT (onset)	Truncation
85	M	29	4	+	+	+	+	+	+	Severe	+	DPT	Truncation
89	M	5	5	+	+	+	−	+	+	Moderate	+	DPaT	Splice site
94	F	10	4	+	+	+	+	+	+	Mild	+	−	Missense
100	M	8 mo.	3	+	+	−	+	−	+	N.A.	−	−	Truncation
83	F	13	7	+	+	+	−	−	+	Mild	−	−	− (Missense in *PCDH19* &*TSPYL4*) &TSP)
67 (non-DS)	F	32	15	−	+	+	−	−	+	Mild	+	−	− (Missense in *PCDH19*)

Mild ID: general/developmental quotient = 50–70;

Moderate ID: general/developmental quotient = 25–50;

Severe ID: general/developmental quotient<25;

DPT: diphtheria, pertussis and tetanus vaccination;

DPaT: DPT vaccines with acellular component of pertussis.

### *SCN1A* Mutations in Patients with Dravet Syndrome (Table 2)

**Figure 1:**
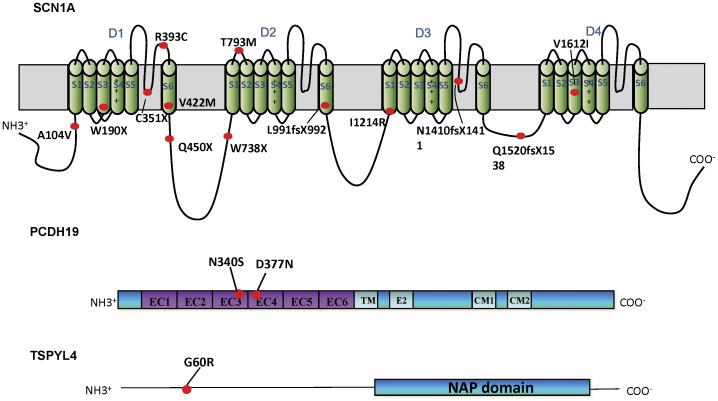
Schematic diagram showing the location of the identified mutations in the three encoded proteins.

Of the 18 DS patients examined, 15 (83%) were identified to have *SCN1A* mutations (Table 1). Six of these mutations were missense mutations, seven were non-sense or frameshift mutations, and two were mutations in intronic splice donor or acceptor sites (Table 2). These mutations spread throughout the whole *SCN1A* gene. Among the 6 missense mutations, 3 missense mutations (c.311C>T, p.A104V; c.1177C>T, p.R393C and c.4834G>A, p.V1612I) have been reported previously [8] and 3 missense *SCN1A* mutations were novel (c.1264G>A, p.V422M; c.2378C>T, p.T793M and c.3641T>G, p.I1214R). For the 7 truncating mutations, 2 (c.1348C>T, p.Q450X and c.569G>A, p.W190X) were reported previously [10], [29] and 5 (c.1053T>A, p.C351X; c.2214G>A, p.W738X; c.2971-2972delCTinsG, p.L991fsX992; c.4229delA, p.N1410fsX1411; c.4558delC, p.Q1520fsX1538) were novel. The two predicted aberrant splicings were resulted from 2 novel mutations in intron 3 (IVS3+3A>C) and intron 21 (IV21+1G>A). Most of the mutations were in D1 and D2 of the SCN1A protein and no mutation were located in S4 voltage sensing segment. The types and locations of different mutations found in the 15 DS patients were summarized in Table 2 and Fig. 1. No *SCN1A* mutation was detected in the coding region of *SCN1A* in the other 3 DS patient. Parental DNAs of 14 mutation positive cases were available for *SCN1A* gene analysis and 1 out of 15 cases were unavailable. Analysis of parental DNA confirmed that 12 cases of mutations were *de novo*, 2 mutations (p.V1612I and p.T793M) were inherited maternally. As the mothers of these 2 patients had the same mutation but remain asymptomatic, the inherited mutations do not fully explain the phenotypes. None of the mutations were found in 100 normal control alleles. Among the 15 DS patients with *SCN1A* mutations, 8 patients (53%) had a history of seizures following vaccinations. Three of these patients had onset of first seizures related to vaccination. The majority (75%) of seizures occurred after DPT vaccinations. There was no history of vaccination-induced seizure for the 3 DS patients with no *SCN1A* mutation.

**Table 2:** Summary of *SCN1A*, *PCDH19* and *TSPYL4* mutations found in 17 patients.

Gene	Case	Dravet syndrome(+/−)	Type ofmutation	Amino acid	Exon	Nucleotide	Location in protein	*De novo*
*SCN1A*	6	+	Frameshift	p.L991fsX992	16	c.2971–2972delCTinsG	S6 of D2	+
	77	+	Frameshift	p.N1410fsX1411	21	c.4229delA	S5–S6 of D3	+
	100	+	Frameshift	p.Q1520fsX1538	24	c.4558delC	D3–D4 loop	+
	53	+	Nonsense	p.Q450X	9	c.1348C>T	D1–D2 loop	+
	71	+	Nonsense	p.W190X	4	c.569G>A	S3 of D1	+
	74	+	Nonsense	p.W738X	13	c.2214G>A	D1–D2 loop	+
	85	+	Nonsense	p.C351X	8	c.1053T>A	S5–S6 of D1	N.A.
	24	+	Splice site	−	Intron 21	IVS21+1G>A	S5–S6 in D3	+
	89	+	Splice site	−	Intron 3	IVS3+3A>C	S2 of D1	+
	40	+	Missense	p.T793M	13	c.2378C>T	S1–S2 of D2	Maternal
	46	+	Missense	p.A104V	2	c.311C>T	N-terminus	+
	65	+	Missense	p.V1612I	25	c.4834G>A	S3 of D4	Maternal
	75	+	Missense	p.R393C	9	c.1177C>T	S5–S6 of D1	+
	76	+	Missense	p.V422M	9	c.1264G>A	S6 of D1	+
	94	+	Missense	p.I1214R	18	c.3641T>G	S1 of D3	+
*PCDH19*	67	−	Missense	p.N340S	1	c.1019A>G	EC3	N.A.
	83	+	Missense	p.D377N	1	c.1129G>A	EC4	+
*TSPYL4*	83	+	Missense	p.G60R	−	c.178G>C	N- to NAP	Maternal

D1–D4: four homologous domains of SCN1A; S1–S6: six transmembrane segments in the four domains of SCN1A; EC: extracellular cadherin domain of PCDH19; NAP: domain of nucleosome assembly protein of TSPYL4.

### *PCDH19* Mutations

One of the *SCN1A*-negative female DS patients (case 83) was identified to have a novel *PCDH19* missense mutation in exon 1 (c.1129G>A, p.D377N) (Table 1, 2). One non-DS female patient (case 67) was identified to have another exon 1 missense mutation (c.1019A>G, p.N340S). She presented with severe phenotype of epilepsy and ID overlapping with the features of DS but the onset of seizure was 15 months. Both missense mutations caused amino acid change in the extracellular domain of *PCDH19* with p.D377N in extracellular cadherin - 4 (EC4) domain and p.N340S in EC3 domain (Fig.1). Parental DNA of case 83 was screened and the mutation was confirmed to be *de novo*. Parental DNA of the other case was unavailable. None of the mutations were found in 100 normal control alleles.

### *TSPYL1* Polymorphisms (Table 3)

From NCBI dbSNP, 30 SNPs were reported in the 1314 bp coding region of *TSPYL1* gene. Among these 30 SNPs, 18 SNPs were missense, 6 were synonymous, 3 were insertions and 3 were frameshifts. The nucleotide changes of the 6 insertions/frameshifts were shown in Table 3. Out of these insertions/frameshifts, 4 resulted in premature stop (rs56100880, rs80099151, rs144384201, rs140644109) and the rest resulted in insertion/deletion of amino acids. The 4 SNPs leading to premature stop were expected to result in loss-of-function. As deleterious variations in *TSPYL1* were identified in the general population, mutation screening in our patients was not performed.

**Table 3:** Six insertion/frameshift SNPs of *TSPYL1* from NCBI dbSNPs.

SNP	Nucleotide change
rs67074252	c.520_521insGGT
rs56100880	c.521_522insGGT
rs80099151	c.522delG; c.522delGinsGGT
rs144384201	c.523_524delGT; c.523_524delGTinsGGT
rs140644109	c.527_528delTG; c.527_528delTGinsGTG
rs78371471	c.528_529insGTG

### *TSPYL4* Variant

The DS patients (case 83) with the *de novo* D377N *PCDH19* mutation was found to have a missense variant in *TSPYL4* gene (c.178G>C, p.G60R) (Fig.1, Table 1, 2). This variant was maternally inherited and the patient’s mother was asymptomatic. The variant was not found in 100 normal control alleles.

### Analyses of Missense and Splice Site Mutations (Table 4)

**Figure 2:**
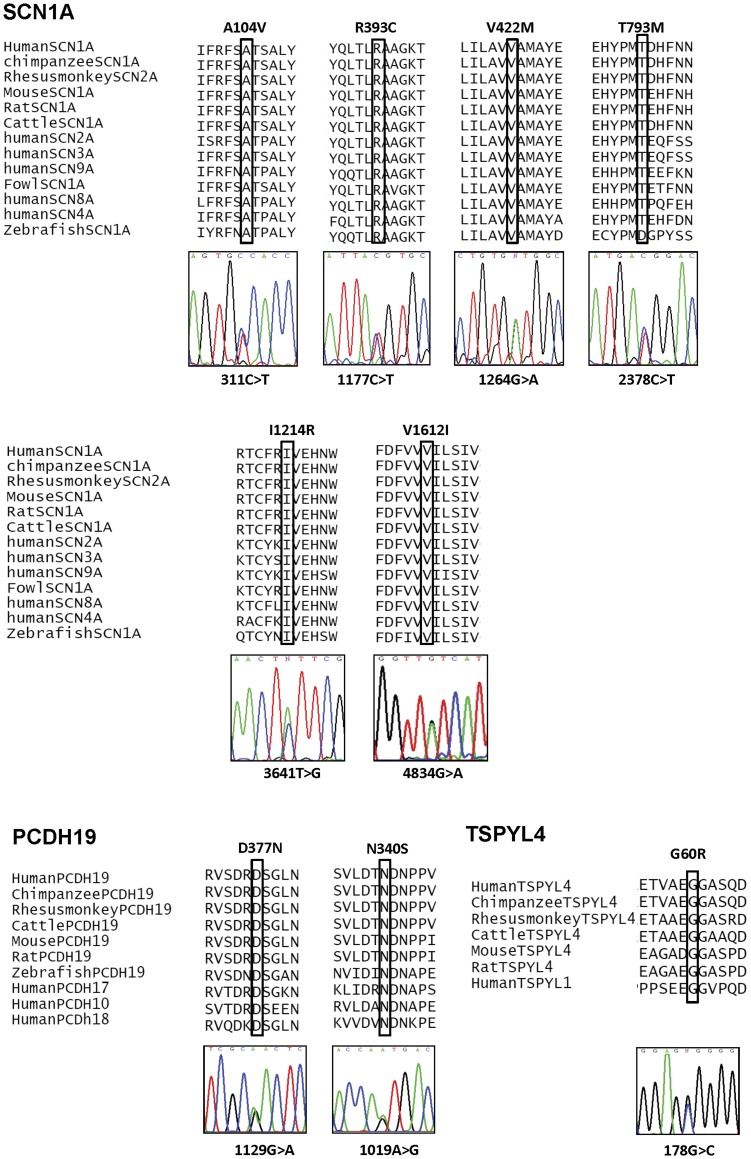
Evolutionary conservations of amino acid substitutions found in SCN1A, PCDH19 and TSPYL4.

The conservation analysis (Fig. 2) in the present study revealed that the 6 missense *SCN1A* mutations, 2 *PCDH19* mutations and 1 *TSPYL4* variant affected highly conserved amino acids. In addition, SIFT analyses predicted that these 9 missense mutations affected protein function. By Align-GVGD analysis (Table 4), the 9 missense mutations were assigned a high class and given an increase likelihood to be deleterious in different extents (p.V1214R, p.A104V, p.R393C and p.G60R were classified as C65; p.N340S was classified as C45; p.V1612I was classified as C25; p.T793M, p.V422M and p.D377N were classified as C15). Automated Splice Site Analyses revealed that both intronic mutations in *SCN1A* could abolish the splice donor sites and caused aberrant splicing.

**Table 4:** Pathogenicity assessment of 9 missense mutations and 2 tolerated amino acid substitutions.

Gene	Change of amino acid in missense mutation	Location in protein	Conservative analysis*	SIFT analysis†	Align-GVGD analysis		
					Grantham Variation	Grantham Deviation	Class‡
*SCN1A*	p.T793M	S1–S2 of D2	+	+	85.08	81.04	C15
	p.A104V	N-terminal	+	+	0.00	65.28	C65
	p.V1612I	S3 of D4	+	+	0.00	28.68	C25
	p.R393C	S5–S6 of D1	+	+	0.00	179.53	C65
	p.V422M	S6 of D1	+	+	0.00	20.52	C15
	p.I1214R	S1 of D3	+	+	0.00	97.59	C65
*PCDH19*	p.N340S	EC3	+	+	0.00	46.24	C45
	p.D377N	EC4	+	+	0.00	23.01	C15
*TSPYL4*	p.G60R	N- to NAP	+	+	0.00	125.13	C65
*SCN1A*	p.D516N	D1–D2 loop	−	−	353.86	0.00	C0
	p.A464P	D1–D2 loop	−	−	110.77	0.00	C0

* For conservative analysis, “+” indicates the amino acid residue is highly conserved and “−” indicated it is less conserved.

† For SIFT analysis, “+” indicates the variant affect protein function and “−” indicated that it is tolerated.

‡ The variants were classified into 7 groups (class 0, class 15, class 25, class 35, class 45, class 55 and class 65) ranging from least likely (class 0) to most likely (class 65) to interfere the protein function (http://agvgd.iarc.fr/classifiers.php).

D1–D4: four homologous domains of SCN1A; S1–S6: six transmembrane segments in the four domains of SCN1A; EC: extracellular cadherin domain of PCDH19; NAP: nucleosome assembly protein domain of TSPYL4.

### *SCN1A* Variations in Patients not Grouped under Dravet Syndrome

No *SCN1A* mutations were found in the 82 non-DS patients. Two variations resulting in amino acid substitutions (p.A464P and p.D516N) were found in 2 out of 82 non-DS patients. Pathogenicity assessment predicted that these two substitutions were tolerated and less likely to interfere protein functions (Table 4).

### Intellectual Disability and *SCN1A* Mutation

**Figure 3:**
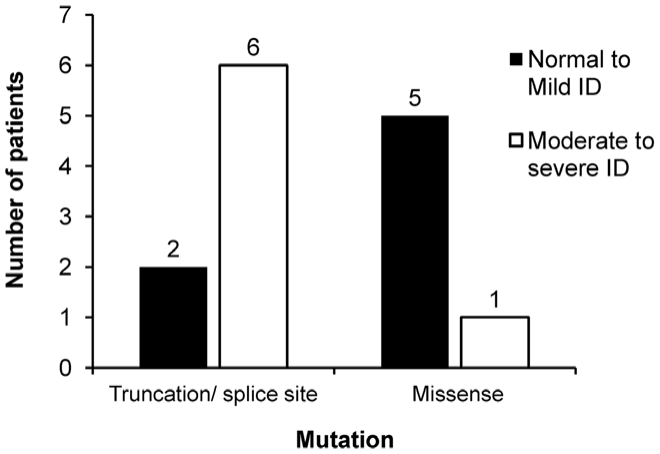
Degree of intellectual disability (ID) of patients with different types of *SCN1A* mutations. The number above the bar chart indicated the number of DS patients in that group. ID: Intellectual disability; Mild ID: general/developmental quotient = 50–70; Moderate ID: general/developmental quotient = 25–50; Severe ID: general/developmental quotient<25.

Seven patients had truncating mutation in *SCN1A* gene, of which 3 (cases 71, 74 and 85) had severe ID, 1 (case 6) had moderate ID, 1 (case 77) had mild ID and 1 (case 53) was normal. The 2 patients (cases 24 and 89) with splice site mutation had moderate ID. On the other hand, 5 patients (cases 40, 46, 75, 76 and 94) with missense mutations had only mild ID and 1 of them (case 65) had moderate ID. Chi-square test showed that there was a relationship between types of *SCN1A* mutation (missense mutation versus truncating/splice site mutation) and the degree of ID (normal to mild ID versus moderate to severe ID) (p<0.05). This indicated that the patients with missense mutations tend to have less severe ID than those with either truncating or splice site mutations (Fig. 3). The 8-month-old DS patient with truncating mutation (case 100) was not included in this analysis as he was too young and long term cognitive outcome was still undetermined.

## Discussion

### *SCN1A* Mutations in 15 Patients with Dravet Syndrome

In this study, we have found 9 truncating and splice site mutations among the 15 *SCN1A* mutations in DS patients. Most truncating mutations terminated the protein at first 2 domains. Early truncating mutations are mostly associated with severe epileptic phenotypes in DS, although in a previous study, they caused only milder epilepsy with GEFS+ and focal seizures in 2 patients [30]. In the present study, all truncating and splice site mutations were *de novo* and linked to DS with severe ID.

In contrast to the truncating and splice site mutations, the association of missense mutations with phenotypic expression of DS patients is more controversial. In this study, we used conservative analysis, SIFT analysis and Align-GVGD to assess the interference of the amino acid substitutions to the protein functions. p.V1612I has been reported in the study of Depienne et al [10] and Herini et al [31] associated with DS. p.R393C has been reported previously in several studies [5], [10], [29], [32] and this mutation was all found in SMEI patients except in the study of Harkin et al [5] who identified the R393C mutation in a patient with myoclonic astatic epilepsy. According to the analysis in Depienne et al. [10] study, both p.R393C and p.V1612I mutations alter highly conserved amino acid residues in the protein. p.R393C substitution involves a very dissimilar change in amino acid residue but p.V1612I substitution only has a slight influence chemically according to Grantham distance determination. However, V1612I substitution alters amino acid in a highly conserved ion transport sequence [10]. In the present study, Align-GVGD analysis showed that p.V1612I substitution can possibly affect protein function (Align-GVGD classified to C25). No mutational analysis of p.A104V has been published previously. We illustrated here that this mutation affects a highly conserved amino acid which is substituted with a chemically dissimilar residues (GD = 65.28) in the N-terminus and very likely (Class C65) to affect SCN1A protein function. Conservative analyses has showed that the three novel mutations (p.V422M, p.T793M and p.I1214R) change highly conserved amino acid residues in the protein and pathogenicity assessment indicated these mutations produce dissimilar changes in amino acids. They change protein function in different extents and are associated with severe phenotypes of the 3 DS patients. By contrast, the 2 substitutions (p.A464P and p.D516N) involve less conserved amino acid residues are predicted to be tolerated and less likely to affect protein function. The two patients with these substitutions were diagnosed as non-DS. These findings illustrated the significance of pathogenicity assessments in elucidating the association of amino acid substitutions with the clinical expression of the DS patients.

Nevertheless, the association between the clinical phenotypes and *SCN1A* mutations is still poorly understood. First, it is unclear how missense mutations can cause an epileptic phenotype similar to truncating mutations. A possible mechanism is haploinsufficiency [10]. Heterozygosity of both loss-of-function truncating and deleterious missense mutations can result in quantitative reduction in normal functioning sodium channel which causes severe phenotypes in DS [10], [33]. Second, both inherited missense mutations (p.V1612I and p.T793M) in the present study were inherited from asymptomatic mothers. In the other reported cases of inherited *SCN1A* mutations [8] including truncating mutations [34], the transmitting parents were also asymptomatic or of mild epileptic phenotypes. Somatic mosaicisms of the parents have been shown to account for this phenomenon [35], [36], [37]. It is also possible that *SCN1A* mutation is not the only factor for the phenotypes in DS patients and other genetic background and environmental factors are involved to produce synergistic effect to the disease. In response to this issue, Depienne et al [11], [35] proposed the possibility of a variable expression of mutation in the brain of asymptomatic parents. They suggested that peripheral mosaicism like blood cells does not always reflect the neuronal mosaicism although they showed that the clinical status of mosaic parents correlated with the percentage (0–85%) of mutation in their blood cells. Meisler and Kearney [33] also proposed several factors such as developmental variability, accumulation of somatic mutation in lifetime, environmental insults and modifier genes which may exacerbate the phenotypes of individuals carrying *SCN1A* mutations. Further investigations on other interacting genetic, developmental and environmental elements will bring more insight into these issues.

Interestingly, here we found no mutation in S4, the voltage sensor region as defined by Kanai et al [38], in contrast to the finding in Zuberi et al [12] that the missense mutations occurred with a higher frequency in S4 region. Besides, there was no preferential localization of mutations in S5–S6 segment, the pore forming region, which was reported previously to have higher frequency of missense mutations in SMEI patients [12], [38]. In agreement with our study, Depienne et al [10] also failed to demonstrate any preferential localization in S5–S6 segment. So, the present study did not support a relationship between the localization of mutation and the severity of the diseases and all *SCN1A* mutations are severe in DS patients.

### Association of *SCN1A* Mutation with Intellectual Disability and Autism Spectrum Disorder

In DS patients, developmental regression in the second year, cognitive deficits as well as behavioral disturbances are common [3]. In the present study, patients with missense mutations tend to be associated with less severe ID than those with either truncating or splice site mutation. These data suggested a possible correlation between the type of *SCN1A* mutation and mental development of DS patients. Riva et al [39] also proposed that *SCN1A* mutation, rather than epilepsy, was responsible for the progressive cognitive impairment. They showed that two DS children carrying *SCN1A* truncating mutations had their cognitive performance continued to deteriorate irrespective of the difference in their epilepsy severities. However, the study of Ragona et al [40] failed to demonstrate a significant difference in the cognitive outcome of 26 cases with regard to the presence and type of *SCN1A* mutation. They showed that the worst cognitive outcome was associated with early appearance of myoclonus and absences. A recent review also suggested that modifier genes present in DS patients may be the factor affecting the cognitive outcome [41]. Additional clinical investigations with large sample sizes will be necessary to elucidate the correlation between cognitive development in DS patients and the types of *SCN1A* mutations.

In the present study, 11 (73%) out of 15 DS patients with *SCN1A* mutation had ASD in the present study. A previous study identified four missense mutation in *SCN1A* gene, one coding mutation in *SCN2A* gene from different autism families [42] and suggested that mutations in the sodium channel genes may play a role in autism susceptibility. O’Roak et al [43] also found a functionally deleterious *SCN1A* missense mutation in a patient with sporadic ASD. These findings indicate that autism may be one of the possible consequences of *SCN1A* mutation. Osaka et al [44] identified a novel *SCN1A* mutations in two patients with both seizures and psychiatric disorders including panic disorder and Asperger syndrome. They suggested that *SCN1A* mutation may contribute to the expression of psychiatric disorders in addition to epileptic seizures and combined effect of different gene variants may determine the ultimate phenotype of patients [33], [42]. Li et al [45] reported that the DS patients with autism had more severe ID [45]. However, in the present study, DS patients with ASD could have mild, moderate or severe grade of ID and there is no association with the degree of ID. Association of *SCN1A* mutation in epilepsy patients with evolving autistic features is an interesting topic for future clinical research.

### Vaccination-induced Seizures or Vaccine Induced Encephalopathy in Patients of Dravet Syndrome with *SCN1A* Mutations

In the present study, 8 DS patients with *SCN1A* mutations had a history of seizures following vaccinations. Our finding elucidated that seizures after vaccinations are common in DS patients (8/18; 44%) and it is also the case in the study of Tro-Baumann et al [46] with 19 (27%) of 70 patients having seizures after vaccination. In contrast, the rate of febrile seizures occurring in general population is much lower (6–9 and 25–34 per 100,000 children) after DPT and MMR vaccines administration [47]. The high rate of vaccination-induced seizures in DS patients with *SCN1A* mutation (53%) may be connected to the genetically determined aberrant functioning of sodium channels. Berkovic et al [47] have also suggested that vaccination is unlikely to be the significant trigger of the encephalopathy in DS patients with *SCN1A* mutation and avoiding vaccination will not prevent the onset of disorders. Further genetic study on patients with vaccination-induced seizures should be considered. Testing for *SCN1A* mutations will be necessary for those infants suspected to have DS in order to provide early management in terms of temperature and infection controls.

### *PCDH19* Mutations in 2 Patients

*PCDH19* is another susceptibility gene for female patients of DS. In the present study, one of the two *SCN1A*-negative female DS patients was identified to have a novel *PCDH19* mutation (p.D377N). Pathogenicity assessment showed that this mutation changes highly conserved amino acids in EC4 domain and affects protein function. This *PCDH19*-positive DS patient shared the same clinical features with other DS patients except the absence of both myoclonic and absence seizures. Besides, this patient had clusters of seizures during febrile episodes which was another distinguishing feature observed in *PCDH19*-positive patients as suggested by Depienne et al [48]. The proportion of *PCDH19* mutation among DS patients (5.6%) in the present study was comparable to Depienne et al. [22] study. Depienne et al [22] suggested that *PCDH19* might contribute significantly to epileptic encephalopathies with clinical spectrum overlapping that of DS. They showed that *PCDH19*-DS and *SCN1A*-DS had many common features including early onset of seizures, association of generalized tonic-clonic and focal seizures, prolonged seizures and developmental regression. But they differed slightly as *PCDH19*-DS might have a later seizure onset, less intractable seizure, absence of status epilepticus, myoclonic jerks/atypical absences and less severe intellectual/language delay.

Another *PCDH19* mutation (p.N340S) was found in a non-DS patient. Both patients with a *PCDH19* mutation shared similar clinical features including the presence of both partial and generalized seizure types, occurrence of status epilepticus and mental retardation. However, the non-DS patients had seizure onset after 1 year (15 months). p.N340S has been reported previously in three studies [22], [49], [50]. In the studies of Depienne et al [22] and Marini et al [49], the patients with p.N340S mutations were diagnosed as DS. However, the sister pair with this mutation in the study of Dibben et al [50] were diagnosed as EFMR. They had seizure onset at 13 and 17 months, refractory seizure and developmental slowing. Patients with *PCDH19* mutations in other studies [15], [16], [48] were also shown to be non-DS with seizure onsets after 1 year. Taken together, in addition to DS patients, *PCDH19* mutation screening should also be considered for patients with clinical features resembling DS with seizure onset after 1 year old.

### *TSPYL4* Variant in 1 Patient

Interestingly, a new *TSPYL4* variant was found in a DS patient with a novel *PCDH19* p.D377N mutation. The amino acid substitution p.G60R occurs in a highly conserved amino acid and is likely to interfere with protein function according to pathogenicity assessment. Nevertheless, it is N-terminal to the conserved nucleosome assembly protein domain. In addition, the DS patient with the *TSPYL4* mutation is also *PCDH19*-positive and his mother carrying the *TSPYL4* mutation is asymptomatic. Besides, the role of *TSPYL4* in epilepsy has not been reported previously. Taken together, the *TSPYL4* variant we identified is unlikely to be pathogenic.
